# Inflammatory biomarkers and sarcopenia in older adults

**DOI:** 10.3389/fragi.2026.1770257

**Published:** 2026-05-25

**Authors:** Ahalya Kanakan, Anup Singh, Sarita Kumari, Surendra Pratap Mishra

**Affiliations:** 1 Geriatric Medicine, Institute of Medical Sciences, Banaras Hindu University, Varanasi, India; 2 Anesthesia (Trauma), Institute of Medical Sciences, Banaras Hindu University, Varanasi, India; 3 Biochemistry, Institute of Medical Sciences, Banaras Hindu University, Varanasi, India

**Keywords:** biomarker, elderly, IL-6, sarcopaenia, TNF-alpha (TNFα)

## Abstract

Chronic inflammation contributes to the pathogenesis of sarcopenia in older adults. This study aimed to evaluate the correlation between inflammatory biomarkers such as C-reactive protein (CRP), tumor necrosis factor-alpha (TNF-α), interleukin-6 (IL-6), and the anabolic hormone insulin-like growth factor-1 (IGF-1) with sarcopenia. A hospital-based case-control study was conducted among patients aged ≥60 years attending the Geriatric Medicine outpatient clinic. Participants were screened using the SARC-F tool and sarcopenia diagnosis was confirmed according to the Asian Working Group for Sarcopenia (AWGS) criteria. Serum levels of CRP, IL-6, TNF-α, and IGF-1 were measured using enzyme-linked immunosorbent assay (ELISA) in 30 sarcopenic cases and 30 age- and sex-matched controls. Group differences were assessed using independent samples t-test or Mann–Whitney U test, and correlations were evaluated using Spearman’s rank correlation coefficient. The mean ages of cases and controls were 67.6 ± 7.5 and 66.1 ± 6.2 years, respectively; females comprised 56.7% of cases and 46.7% of controls. Mean CRP (5.58 ± 2.47 mg/L vs. 2.98 ± 3.00 mg/L; *p* = 0.001) and TNF-α levels (176.66 ± 163.10 pg/mL vs. 45.10 ± 89.14 pg/mL; *p* < 0.001) were significantly higher in sarcopenic cases compared to controls. No statistically significant differences were found for IL-6 or IGF-1 levels. Increased CRP and TNF-α levels are associated with sarcopenia in older adults, suggesting a role of systemic inflammation in its pathogenesis. IGF-1, an anabolic hormone known to be influenced by inflammation, did not differ significantly between groups. Larger studies with rigorous exclusion of confounders are needed to validate these findings and clarify the role of inflammatory processes in sarcopenia.

## 1 Introduction

Sarcopenia is a progressive and generalized skeletal muscle disorder characterized by loss of muscle mass, strength, and/or physical performance, and is a major contributor to frailty, increasing the risk of falls, fractures, disability, hospitalization, long-term care dependency, and mortality (Walrand et al., 2011; Lang et al., 2010). Increase in life expectancy has led to a growing population of older adults, accompanied by a rise in chronic age-related morbidities (World Health Organization, 2025). While conditions such as diabetes, hypertension, cardiovascular disease, cerebrovascular disorders, and malignancies often receive considerable clinical attention, age-related changes in body composition, particularly sarcopenia, remain underdiagnosed and inadequately managed in many clinical settings, especially in low- and middle-income countries.

Sarcopenia is now recognized as a geriatric syndrome involving a decline in muscle mass, accompanied by impaired muscle function (Chen et al., 2020). However, despite its significant clinical implications, sarcopenia remains underdiagnosed and inadequately managed. According to a systematic review, the prevalence of sarcopenia varies widely, ranging from 1% to 29% among community-dwelling older adults, 14%–33% in long-term care residents, and approximately 10% in hospitalized older adults (Cruz-Jentoft et al., 2014). The co-occurrence of sarcopenia and obesity, termed sarcopenic obesity, is particularly common in adults aged 65 years and older, with prevalence influenced by comorbidities ((Kumari et al., 2022). Skeletal muscle mass begins to decline gradually from approximately age 30 at a rate of 0.1%–0.5% per year, with the rate accelerating after age 65 (Hughes et al., 2002). This age-related muscle loss, together with decreased physical performance, poses a major public health challenge, contributing to increased healthcare utilization and mortality (Morley et al., 2014). Given that women generally have lower baseline muscle mass and longer life expectancy than men, sarcopenia represents a greater health concern for older women (Roubenoff and Hughes, 2000).

The etiology of sarcopenia is multifactorial, including physical inactivity, malnutrition, hormonal changes, altered cytokine profiles, neuromuscular degeneration, genetic factors, and chronic low-grade inflammation associated with aging and comorbid conditions (Walrand et al., 2011; Dhillon and Hasni, 2017; Kim and Choi, 2013).

Among the multiple pathophysiological pathways implicated in sarcopenia including neuromuscular junction degeneration, hormonal decline, mitochondrial dysfunction, and oxidative stress chronic low-grade systemic inflammation (“inflamm-aging”) has emerged as a particularly important and modifiable contributor. Inflammatory mediators such as CRP, TNF-α, and IL-6 are readily quantifiable through standardized ELISA-based assays, making them accessible biomarker candidates for clinical settings, particularly in resource-limited environments. Furthermore, the anabolic hormone IGF-1, which may be downregulated in inflammatory states, provides an additional dimension linking inflammation to sarcopenia pathogenesis. While biomarkers from other systems (e.g., C-terminal agrin fragment for neuromuscular junction integrity, myostatin for muscle turnover, GDF-15 for mitochondrial stress) are emerging as promising candidates, they remain less well-characterized in diverse populations and less readily available in routine clinical practice (Casati et al., 2019; Popescu et al., 2026).

Early diagnosis and intervention are critical to improving outcomes. However, differentiating physiological muscle decline from pathological sarcopenia remains challenging due to nonspecific clinical features. This underscores the need for reliable, cost-effective biomarkers to improve early detection and guide clinical management. Previous studies have reported positive associations between sarcopenia and inflammatory markers such as C-reactive protein (CRP), interleukin-6 (IL-6), and tumor necrosis factor-alpha (TNF-α), alongside a negative association with the anabolic hormone insulin-like growth factor-1 (IGF-1), which may be downregulated in inflammatory states (Bian et al., 2020; Bian et al., 2017; Ying et al., 2022; Taaffe et al., 2000).

To our knowledge, the correlation between these biomarkers and sarcopenia has not been comprehensively studied in the Indian older adult population. This study aims to fill this gap by assessing the relationship between sarcopenia and selected inflammatory and anabolic biomarkers in older adults attending a tertiary care hospital in India.

## 2 Methods

### 2.1 Study design

This hospital-based case-control study was conducted among older adults aged ≥60 years attending the Geriatric Outpatient Department of a tertiary care hospital in northeastern India.

### 2.2 Participants

A total of 60 participants (30 sarcopenic cases and 30 non-sarcopenic controls), including both males and females, were recruited consecutively from the same outpatient clinic. Written informed bilingual consent was obtained from all participants. Cases and controls were age and sex matched to minimize confounding. All participants underwent thorough clinical assessment and sarcopenia screening as detailed below.

### 2.3 Sample size calculation

Based on prior studies assessing inflammatory biomarkers in sarcopenia and methodological standards for moderate effect sizes (Cohen’s d ≈ 0.7), a sample size of at least 26 participants per group was calculated to achieve 80% power at α = 0.05 (Bian et al., 2020). The primary outcome for the power analysis was the between group difference in serum CRP levels, based on effect sizes reported in the reference study. A two-tailed independent samples t-test was used for the calculation. To allow for potential dropouts, 30 participants per group were enrolled.

### 2.4 Inclusion and exclusion criteria

In this study, the inclusion criteria comprised older adults aged 60 years and above who were able to stand and walk unaided and were diagnosed with sarcopenia according to the Asian Working Group for Sarcopenia (AWGS 2019) criteria (Chen et al., 2020). Controls included participants with similar age characteristics but without sarcopenia. Exclusion criteria were designed to remove confounding factors that could affect muscle mass or inflammatory status. These included individuals younger than 60 years, those with malignancy, bedridden patients due to serious illness, subjects with inadequately managed severe endocrine disorders, infectious diseases, autoimmune diseases or inflammatory arthritis, and those with a history of mental illness. Additionally, participants unable to perform strength or physical performance assessments were excluded to ensure reliable diagnostic measurements.

### 2.5 Sarcopenia screening and diagnosis

Screening was performed using the validated SARC-F questionnaire (Malmstrom and Morley, 2013), assessing strength, assistance walking, rising from a chair/bed, climbing stairs, and history of falls. A score ≥4 suggests sarcopenia risk. Diagnosis followed AWGS 2019 criteria: low muscle mass [<7.0 kg/m^2^ for men, <5.7 kg/m^2^ for women by Bioelectrical Impedance Analysis (BIA)] plus either low muscle strength (handgrip strength <28 kg men, <18 kg women) or low physical performance (6-m gait speed <1.0 m/s, Short Physical Performance Battery ≤9, or 5-time chair stand ≥12 s).

Muscle mass was measured using the InBody 270 BIA device. Handgrip strength was assessed with a Jamar hydraulic hand dynamometer. Physical performance was evaluated by the 6-m walking test.

### 2.6 Demographic and clinical data collection

A pre-designed case report form captured demographic data, marital status, lifestyle factors (smoking, alcohol, tobacco use), and medical history (diabetes, hypertension, chronic obstructive pulmonary disease). Smoking was coded as “current smoker” *versus* “non-smoker” (including never-smokers and ex-smokers who had quit >1 year prior). Alcohol use was coded as “current user” (any alcohol consumption within the past year) *versus* “non-user.” Tobacco chewing was coded as “current user” *versus* “non-user.” Comorbidities (diabetes, hypertension, COPD) were coded as binary variables based on documented clinical diagnosis or current use of disease-specific medications. Height and weight were measured using a standard stadiometer and calibrated scale to calculate body mass index (BMI).

### 2.7 Laboratory assessments

All blood samples were collected in the morning (between 8:00 a.m. and 10:00 a.m.) after an overnight fast, with the participant at rest in a seated position for at least 10 min prior to venipuncture. Five milliliters of venous blood were drawn via standard venipuncture technique into EDTA tubes for routine hematological (including hemoglobin) and plain (red-top) vacutainer tubes for biochemical analysis (including serum albumin) on the same day. An additional 5 mL of blood was collected into plain (red-top) vacutainer tubes for biomarker analysis.

### 2.8 Biomarker quantification

The 5 mL blood sample was centrifuged at 3,000 rpm (approximately 1,500 × g) for 15 min at 4 °C; serum was aliquoted and supplemented with a protease inhibitor cocktail (Sigma-Aldrich, Cat. No. P8340) at a dilution of 1:100 (v/v) as per the manufacturer’s recommended protocol to prevent protein degradation and stored at −80 °C until analysis. ELISA kits were used to quantify CRP (Thermo Fisher, Cat. No. EHCRP), IL-6 (R&D Systems, Cat. No. D6050), TNF-α (Abcam, Cat. No. ab181421), and IGF-1 (RayBiotech, Cat. No. ELH-IGF1-1), following manufacturers’ protocols. Samples were assayed in duplicate, and absorbance was read with a microplate reader. The intra-assay and inter-assay coefficients of variation (CVs) were: CRP (<8% and <10%), IL-6 (<6% and <9%), TNF-α (<7% and <10%), and IGF-1 (<5% and <8%), as reported by the respective manufacturers.

### 2.9 Statistical analysis

Descriptive statistics summarized demographic and clinical characteristics: means ± standard deviations for normally distributed data, medians with interquartile ranges for skewed variables, and percentages for categorical data. Normality of continuous variables was assessed using the Shapiro–Wilk test. Age, BMI, hemoglobin, albumin, and grip strength were normally distributed, whereas CRP, IL-6, TNF-α, IGF-1, SMI, and gait speed showed non-normal distributions. Group differences were assessed using parametric (t-test) or non-parametric (Mann–Whitney U) tests based on data normality. Spearman’s rank correlation was selected over Pearson’s correlation because the majority of biomarker variables showed non-normal, right-skewed distributions. Spearman’s rank correlation tested associations between biomarkers and sarcopenia parameters, including skeletal muscle index (SMI), handgrip strength, and gait speed. Binary logistic regression adjusting for age, sex, and BMI was performed to assess the independent association of biomarkers with sarcopenia. In these analyses, each biomarker (CRP, TNF-α, IL-6, and IGF-1) was entered as a continuous variable; the resulting odds ratios (ORs) therefore reflect the change in odds of sarcopenia per one-unit increase in the respective biomarker concentration. Receiver operating characteristic (ROC) curves were constructed for significant biomarkers, and optimal cut-off values were determined using Youden’s index. Statistical analyses were performed using IBM SPSS version 23 (IBM Corp., Armonk, NY, United States). A *p*-value < 0.05 was considered statistically significant. All tests were two-tailed. Given the exploratory nature of this study, formal correction for multiple comparisons was not applied; however, we note that the primary findings (CRP and TNF-α) would remain significant after Bonferroni correction, and this is acknowledged as a limitation.

## 3 Results

### 3.1 Patient characteristics

The baseline characteristics of study participants are summarized in Table 1. There was no significant difference in age between cases and controls (p = 0.405). The sarcopenic group exhibited significantly lower body mass index (BMI), hemoglobin (Hb), and serum albumin levels compared to controls. Although the prevalence of comorbidities such as diabetes, hypertension, and chronic obstructive pulmonary disease (COPD) was higher among cases, these differences were not statistically significant. Grip strength was significantly lower in the sarcopenic group (p < 0.001), and both SMI (p < 0.001) and gait speed (p < 0.001) were significantly lower in cases compared to controls.

**TABLE 1:** Baseline characteristics of study participants.

Variable	Cases (n = 30)	Controls (n = 30)	p-value
Gender, Male:Female	13 (43.3%)/17 (56.7%)	16 (53.3%)/14 (46.7%)	0.438
Age (years), mean ± SD	67.63 ± 7.56	66.13 ± 6.21	0.405
BMI (kg/m^2^), mean ± SD	22.7 ± 4.6	27.6 ± 4.7	<0.001*
Addictions			
Smoking	4	0	0.038*
Alcohol use	6	1	0.045*
Tobacco chewing	16	3	<0.001*
Comorbidities			
Diabetes	7	5	0.527
Hypertension	5	9	0.229
COPD	3	1	0.308
SMI (kg/m^2^), mean ± SD	5.2 ± 0.9	6.9 ± 0.9	<0.001*
Men	5.89 ± 0.9	7.6 ± 0.5	​
Women	4.65 ± 0.6	6.05 ± 0.4	​
Handgrip strength (kg), mean ± SD	9.63 ± 6.00	21.6 ± 10.9	<0.001*
Men	14.8 ± 4.8	28.9 ± 8.9	​
Women	5.65 ± 3.23	13.2 ± 5.6	​
Gait speed (m/s), mean ± SD	0.79 ± 0.20	1.10 ± 0.25	<0.001*
SARC-F score, mean ± SD	5.2 ± 1.1	1.8 ± 1.28	<0.001*
Hemoglobin (g/dL), mean ± SD	10.6 ± 1.2	11.5 ± 1.3	0.030*
Men	11.3 ± 1.0	12.2 ± 1.7	​
Women	10.1 ± 1.2	10.7 ± 1.4	​
Albumin (g/dL), mean ± SD	3.48 ± 0.45	4.12 ± 0.47	<0.001*

Significant p-values (p < 0.05) are marked with an asterisk (*).

BMI, body mass index; COPD, chronic obstructive pulmonary disease; SMI, skeletal muscle index; SARC-F, strength, Assistance in walking, Rise from a chair, Climb stairs, and Falls questionnaire; SD, standard deviation.

### 3.2 Biomarkers level

Table 2 displays the comparison of inflammatory and anabolic biomarkers between cases and controls. Serum levels of C-reactive protein (CRP) and tumor necrosis factor-alpha (TNF-α) were significantly elevated in sarcopenic subjects (p < 0.001 for both). Conversely, differences in interleukin-6 (IL-6) and insulin-like growth factor-1 (IGF-1) levels did not reach statistical significance.

**TABLE 2:** Comparison of serum biomarkers between sarcopenic cases and controls.

Biomarker	Cases (n = 30) Median (IQR)	Controls (n = 30) Median (IQR)	p-value
CRP (mg/L)	5.35 (4.10–7.20)	2.15 (1.17–3.62)	<0.001*
IL-6 (pg/mL)	9.96 (1.77–53.76)	8.82 (1.64–13.79)	0.387
TNF-α (pg/mL)	79.74 (56.84–330.28)	10.30 (2.19–62.63)	<0.001*
IGF-1 (ng/mL)	0.056 (0.011–0.678)	0.012 (0.010–0.068)	0.141

Significant p-values (p < 0.05) are marked with an asterisk (*).

CRP, C-reactive protein; IL-6, Interleukin-6, TNF-α, Tumor Necrosis Factor-alpha; IGF-1, Insulin-like Growth Factor 1; IQR, interquartile range.

### 3.3 Correlation

Spearman’s rank correlation coefficients between biomarkers and sarcopenia components are illustrated in Table 3. CRP levels correlated inversely with both skeletal muscle index (r = −0.462, *p* < 0.001) and handgrip strength (r = −0.435, p < 0.001), and with gait speed (r = −0.312, p = 0.015). Similarly, TNF-α showed an inverse correlation with SMI (r = −0.397, *p* = 0.0016), though its correlations with grip strength (r = −0.211, *p* = 0.105) and gait speed (r = −0.189, p = 0.148) were not statistically significant.

**TABLE 3:** Spearman correlation between serum biomarkers and sarcopenia components.

Parameter	Correlation coefficient (r)	p-value
SMI and CRP	−0.462	<0.001
Grip strength and CRP	−0.435	<0.001
Gait speed and CRP	−0.312	0.015
SMI and TNF-α	−0.397	0.0016
Grip strength and TNF-α	−0.211	0.105
Gait speed and TNF-α	−0.189	0.148
SMI and IL-6	−0.118	0.369
Grip strength and IL-6	−0.128	0.330
Gait speed and IL-6	−0.094	0.476
SMI and IGF-1	−0.087	0.507
Grip strength and IGF-1	−0.060	0.651
Gait speed and IGF-1	−0.073	0.579

CRP, C-reactive protein; IL-6, Interleukin-6, TNF-α, Tumor Necrosis Factor-alpha; IGF-1, Insulin-like Growth Factor 1; SMI, skeletal muscle index.

### 3.4 Logistic regression and ROC analysis

**FIGURE 1:**
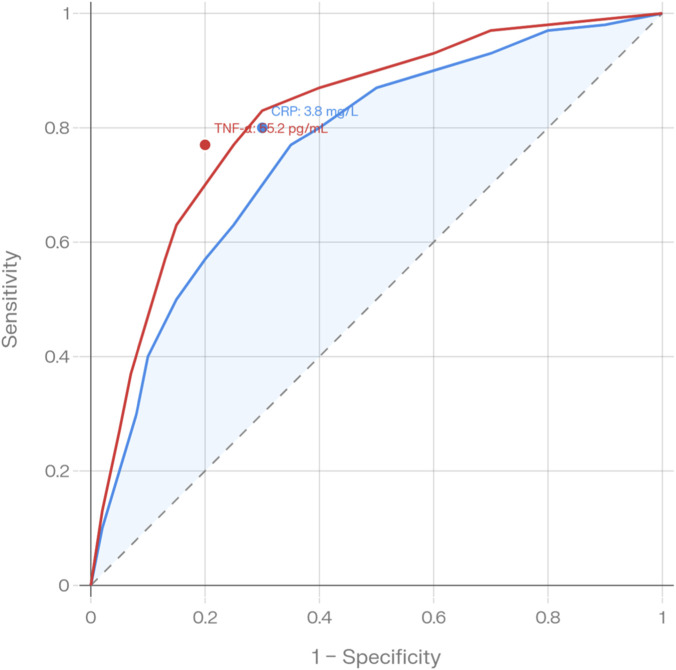
Receiver operating characteristic (ROC) curves for sarcopenia diagnosis using CRP and TNF-α. The curve for CRP (AUC = 0.78, 95% CI: 0.66–0.90, p < 0.001) and TNF-α (AUC = 0.82, 95% CI: 0.71–0.93, p < 0.001) are shown. Optimal cut-off values were determined by Youden’s index: CRP ≥3.8 mg/L (sensitivity 80.0%, specificity 70.0%) and TNF-α ≥ 55.2 pg/mL (sensitivity 76.7%, specificity 80.0%). The diagonal dashed line represents the reference line of no discrimination (AUC = 0.50). AUC, area under the curve; CI, confidence interval; CRP, C-reactive protein; TNF-α, tumour necrosis factor-alpha.

Binary logistic regression adjusting for age, sex, and BMI demonstrated that both CRP and TNF-α were independently associated with sarcopenia (Table 4). Each biomarker was modelled as a continuous variable; accordingly, the ORs represent the change in odds of sarcopenia per one-unit increase in biomarker concentration. CRP (adjusted OR = 1.45, 95% CI: 1.08–1.94, p = 0.013) and TNF-α (adjusted OR = 1.01, 95% CI: 1.00–1.02, p = 0.008) remained significantly associated with sarcopenia after adjustment. The difference in the magnitude of these ORs is attributable to the different units of measurement: CRP is expressed in mg/L (such that an OR of 1.45 reflects the odds change per 1 mg/L increment), whereas TNF-α is expressed in pg/mL (such that the OR of 1.01 reflects the odds change per 1 pg/mL increment, with TNF-α values ranging up to several hundred pg/mL in this cohort). IL-6 (adjusted OR = 1.00, 95% CI: 0.98–1.03, p = 0.672) and IGF-1 (adjusted OR = 0.87, 95% CI: 0.52–1.46, p = 0.598) were not significantly associated with sarcopenia. ROC analysis revealed moderate discriminatory ability for both biomarkers (Table 5 and Figure 1). For CRP, the area under the curve (AUC) was 0.78 (95% CI: 0.66–0.90, p < 0.001), with an optimal cut-off of 3.8 mg/L yielding sensitivity of 80.0% and specificity of 70.0%. For TNF-α, the AUC was 0.82 (95% CI: 0.71–0.93, p < 0.001), with an optimal cut-off of 55.2 pg/mL yielding sensitivity of 76.7% and specificity of 80.0%.

**TABLE 4:** Binary logistic regression for sarcopenia (adjusted for age, sex, and BMI).

Biomarker	Adjusted OR	95% CI	p-value
CRP (mg/L)	1.45	1.08–1.94	0.013
TNF-α (pg/mL)	1.01	1.00–1.02	0.008
IL-6 (pg/mL)	1.00	0.98–1.03	0.672
IGF-1 (ng/mL)	0.87	0.52–1.46	0.598

OR, odds ratio; CI, Confidence interval. Model adjusted for age, sex, and body mass index.

**TABLE 5:** ROC analysis of significant biomarkers for sarcopenia screening.

Biomarker	AUC (95% CI)	Optimal cut-off	Sensitivity (%)	Specificity (%)
CRP (mg/L)	0.78 (0.66–0.90)	3.8	80.0	70.0
TNF-α (pg/mL)	0.82 (0.71–0.93)	55.2	76.7	80.0

AUC, area under the curve; CI, Confidence interval. Optimal cut-off determined by Youden’s index.

## 4 Discussion

Sarcopenia is a multifactorial syndrome associated with adverse health outcomes, including frailty, increased morbidity, and mortality in older adults. Understanding its underlying characteristics and pathogenesis is crucial for prevention and effective management. This study aimed to explore contributing factors and specifically assess the relationship between inflammatory biomarkers and sarcopenia in an older Indian population.

In our cohort, participants predominantly aged 60–70 years were well matched by age and sex between sarcopenic and control groups. Consistent with prior research, the prevalence of sarcopenia was slightly higher in women (56.7%) than men (43.3%). This aligns with community-based studies reporting marginally higher rates among females, potentially related to differences in muscle mass and hormonal factors (Wang et al., 2016; Yamada et al., 2013).

### 4.1 Relationship between inflammatory markers and sarcopenia components

Given the multifactorial nature of sarcopenia and its diagnosis based on skeletal muscle index (SMI), handgrip strength, and physical performance per AWGS criteria, we examined associations between these indices and inflammatory biomarkers. Our findings confirm that serum TNF-α and CRP are significantly correlated with individual sarcopenia components, exhibiting inverse relationships with muscle mass and strength.

CRP demonstrated a significant negative correlation with both SMI and handgrip strength, as well as with gait speed, implicating systemic inflammation in the pathogenesis of sarcopenia. This is consistent with studies such as Stenholm et al. (2008), who observed elevated CRP associated with reduced grip strength, and the Taichung Community Health Study–Elderly cohort, which linked high-sensitivity CRP with sarcopenic obesity in males (Yang et al., 2015). A meta-analysis similarly confirmed associations between CRP and impaired muscle strength, but mostly not with muscle mass (Shokri-Mashhadi et al., 2021). Our observation of an inverse CRP–SMI correlation, which contrasts with some meta-analytic findings, may reflect population-specific characteristics—including the higher prevalence of malnutrition, chronic low-grade inflammation, and distinct nutritional and socioeconomic profiles in Indian older adults—as well as differences in body composition assessment methodology (BIA in our study *versus* DXA in most Western cohorts).

Serum TNF-α levels correlated inversely with SMI but not significantly with grip strength or gait speed. TNF-α is a well-recognized catabolic cytokine implicated in muscle protein degradation mediated by complex systemic pathways. The differential association of TNF-α with muscle mass but not with functional measures (strength and physical performance) may be explained by the fact that TNF-α primarily drives muscle protein breakdown through the ubiquitin–proteasome pathway, directly affecting muscle mass, whereas muscle strength and physical performance are additionally influenced by neural factors, motor unit recruitment, fiber-type composition, and motivation—factors less directly modulated by circulating TNF-α levels. Furthermore, the relatively small sample size may have limited statistical power to detect moderate correlations with these functional parameters. Its circulating levels can vary widely based on factors including age, sex, leukocyte counts, lipid profiles, and genetic predisposition (Haddy et al., 2005; Rall et al., 1996), which may explain inter-individual variability observed in our cohort. Experimental evidence from rodent models confirms the role of TNF-α in proteolysis and muscle atrophy, although direct *in vitro* effects are less conclusive, supporting indirect systemic mechanisms (Goodman, 1991).

Although IL-6 demonstrated negative, yet nonsignificant correlations with sarcopenia indices, previous studies have reported elevated IL-6 levels in sarcopenic individuals, highlighting its putative role as a pro-inflammatory mediator (Bian et al., 2017; Ying et al., 2022). Notably, IL-6 has a well-characterized pleiotropic role: when chronically elevated by adipose tissue and immune cells, it acts as a pro-inflammatory cytokine promoting muscle catabolism through the JAK-STAT3 pathway; conversely, during acute exercise, it is transiently released by skeletal muscle as a myokine, exerting anti-inflammatory and metabolic benefits. This dual nature may explain the inconsistent associations observed between IL-6 and sarcopenia components across studies, as the net effect depends upon the context and chronicity of its elevation (Dupont et al., 2023).

Regarding IGF-1, an anabolic hormone affected indirectly by inflammation, no significant associations with sarcopenia were observed despite levels being generally lower than established references. This contrasts with findings from Bian et al., who reported an independent relationship between reduced IGF-1 and muscle loss in a Chinese cohort (Bian et al., 2020). The complexity of hormonal regulation and cross-sectional design limitations may underlie these discrepancies.

### 4.2 Role of body mass index and nutritional status

Body mass index (BMI) differed significantly between sarcopenic cases and controls in our study. Most controls (20 of 30) had a BMI above 25 kg/m^2^, primarily in the 25–29.9 range, whereas the majority of sarcopenic participants had a BMI below 25 kg/m^2^. This pattern aligns with findings from prior cross-sectional studies reporting lower BMI among sarcopenic individuals compared to controls (Rong et al., 2018). Furthermore, a single-center cross-sectional study by Nakanishi et al. in Japanese patients with type 2 diabetes mellitus demonstrated the utility of BMI as a screening marker for sarcopenia, establishing an optimal cut-off of 24 kg/m^2^ and suggesting BMI’s diagnostic performance is comparable to that of usual gait speed (Nakanishi et al., 2021). Taken together, these observations indicate that low BMI alone does not necessarily imply sarcopenia; therefore, individuals with BMI in the normal range should also be screened to avoid underdiagnosis.

Both obesity and sarcopenia pose critical health challenges in older adults. Obesity promotes a pro-inflammatory milieu via the accumulation of pro-inflammatory macrophages in adipose tissue and dysregulated release of adipokines and cytokines. Additionally, infiltration of adipose tissue into skeletal muscle leads to intramuscular lipid accumulation, impairing mitochondrial function and stimulating production of pro-inflammatory myokines (Bosma et al., 2013). These myokines can further exacerbate adipose tissue inflammation, creating a deleterious feedback loop that contributes to sarcopenic obesity development.

Compared with normative data, handgrip strength in our population was markedly reduced. Control males and females had mean grip strengths of 28.8 ± 8.7 kg and 13.2 ± 5.6 kg, respectively, whereas sarcopenic males and females exhibited 14.8 ± 4.8 kg and 5.7 ± 3.2 kg, respectively. Similar trends were documented in hospital-based studies from northern and southern India (Gunasekaran et al., 2016; Sundarakumar et al., 2022), reporting lower grip strengths than international benchmarks. Korean population studies and pooled datasets from multiple Asian cohorts suggest higher cutoff values for sarcopenia diagnosis (e.g., 28.6 kg men, 16.4 kg women) than those observed in Indian populations (Lee et al., 2019; Auyeung et al., 2020). The AWGS 2019 consensus endorses cutoffs of <28 kg for men and <18 kg for women (Chen et al., 2020). Phenotypic, nutritional, socioeconomic, and environmental differences may justify redefining these thresholds based on local normative data to improve diagnostic accuracy.

In our cohort, serum albumin levels were significantly lower in sarcopenic participants (3.48 ± 0.45 g/dL) than controls (4.12 ± 0.47 g/dL). Previous studies correlate low serum albumin (<3.8 g/dL) with increased disability and mortality in older adults, attributing this decline partly to age-related changes in albumin distribution, synthesis inhibition during inflammatory responses, and enhanced protein degradation (Baumgartner et al., 1996; Rothschild et al., 1988). Hence, hypoalbuminemia may reflect chronic low-grade inflammation contributing to sarcopenia pathogenesis.

Our data revealed significant inverse correlations of serum CRP with muscle mass, grip strength, and gait speed, and TNF-α with muscle mass. The ROC analyses demonstrated moderate discriminatory ability for CRP (AUC = 0.78) and TNF-α (AUC = 0.82), and logistic regression confirmed their independent association with sarcopenia after adjusting for age, sex, and BMI. While these findings suggest potential clinical utility as adjunctive screening biomarkers, the moderate correlation strengths (r = −0.397 to −0.462) and AUC values indicate that these biomarkers are unlikely to be sufficient as standalone diagnostic tools for sarcopenia. Their clinical translation would likely require integration into a composite diagnostic approach incorporating bioelectrical impedance analysis, imaging, muscle function tests, and a multi-system biomarker panel.

### 4.3 Biomarkers across other pathophysiological pathways

While this study focused on inflammatory biomarkers, sarcopenia involves multiple interconnected pathophysiological systems, and emerging evidence supports the role of biomarkers from several other pathways. From a neuromuscular perspective, the C-terminal agrin fragment (CAF) has been identified as a marker of neuromuscular junction (NMJ) degradation, with elevated serum levels associated with sarcopenia and denervation-related muscle atrophy (Drey et al., 2013; Pratt et al., 2024; Leeuwenburgh et al., 2024). Additionally, neurofilament light chain (NfL) is gaining attention as a marker of neurogenic muscle loss and motor neuron degeneration, with recent studies demonstrating associations between elevated NfL and reduced muscle mass and function (Pratt et al., 2022; Ladang et al., 2023; Margoca-Contreras et al., 2022; Tari et al., 2023). The core SNARE protein SNAP-25 has been proposed as a promising biomarker linking NMJ dysfunction to sarcopenia through its role in neuromuscular signal transduction. In the hormonal domain, age-related declines in testosterone, dehydroepiandrosterone sulfate (DHEAS), and growth hormone mediated through reduced IGF-1 signaling contribute to anabolic resistance and impaired muscle regeneration (Giannoulis et al., 2012; Bian et al., 2020). At the mitochondrial level, markers of mitochondrial dysfunction such as growth differentiation factor-15 (GDF-15), advanced glycation end-products (AGEs), and dysregulated PGC-1α signaling reflect the role of oxidative stress and impaired energy metabolism in sarcopenia pathogenesis (Kamper et al., 2024; Lim et al., 2024). Muscle-specific biomarkers including myostatin, brain-derived neurotrophic factor (BDNF), and procollagen type III N-terminal peptide (P3NP) provide additional insights into muscle protein turnover and regeneration capacity; notably, lower circulating BDNF has been associated with reduced muscle strength and mass in older adults (Pratt et al., 2025; da Costa Teixeira et al., 2023; Hsu et al., 2023; Huang et al., 2025). Future studies should consider integrating biomarkers from these multiple pathways into composite panels to improve screening accuracy and provide a more comprehensive biological characterization of sarcopenia.

Our findings, considered alongside the broader body of literature, provide preliminary evidence that inflammatory biomarkers such as CRP and TNF-α may have potential as adjunctive measures in sarcopenia assessment. However, the viability of biomarker-based screening is likely dependent upon a composite panel approach integrating markers from multiple pathophysiological pathways to give a composite risk score. The specificity and sensitivity of these markers require validation in larger, multicentric cohorts incorporating confounder adjustments. Longitudinal studies are essential to establish causality and explore the temporal sequence of inflammation and muscle decline.

This study’s single-center, cross-sectional design and modest sample size limit the generalizability and causal inferences. Potential confounders influencing inflammatory biomarker levels cannot be fully excluded. The absence of formal correction for multiple comparisons, and the limited power for comprehensive multivariable analyses, represent additional methodological limitations. Nonetheless, to our knowledge, this is the first Indian study evaluating sarcopenia-inflammation correlations, providing foundational data for future research.

This study demonstrates a significant association between elevated inflammatory biomarkers specifically C-reactive protein (CRP) and tumor necrosis factor-alpha (TNF-α) and sarcopenia in older adults. These findings support the role of systemic low-grade inflammation in the pathogenesis of sarcopenia and highlight the potential utility of CRP and TNF-α as adjunctive biomarkers for its diagnosis. However, further larger-scale prospective studies are warranted to validate these markers, clarify their clinical relevance, and establish population-specific diagnostic thresholds. Understanding the complex interplay between inflammation, muscle loss, and aging may facilitate earlier identification and more targeted interventions to mitigate sarcopenia’s impact on health outcomes in elderly populations.

## Data Availability

The raw data supporting the conclusions of this article will be made available by the authors, without undue reservation.
